# Influence of diabetes on short-term outcome after major hepatectomy: an underestimated risk?

**DOI:** 10.1186/s12893-020-00971-w

**Published:** 2020-11-30

**Authors:** Alexander Fischer, Juri Fuchs, Christos Stravodimos, Ulf Hinz, Adrian Billeter, Markus W. Büchler, Arianeb Mehrabi, Katrin Hoffmann

**Affiliations:** 1 Department of General and Transplant Surgery, University Hospital Heidelberg, Ruprecht-Karls-University, Im Neuenheimer Feld 110, 69120 Heidelberg, Germany; 2 Department of General and Visceral Surgery, Municipal Hospital Karlsruhe, Moltkestrasse 90, 76133 Karlsruhe, Germany

**Keywords:** Major hepatectomy, Risk factors in liver surgery, Diabetes in liver surgery, Overweight in liver resection, Metabolism, Liver resection, Liver cancer, Liver metastasis

## Abstract

**Background:**

Patient-related risk factors such as diabetes mellitus and obesity are increasing in western countries. At the same time the indications for liver resection in both benign and malignant diseases have been significantly extended in recent years. Major liver resection is performed more frequently in a patient population of old age, comorbidity and high rates of neoadjuvant chemotherapy. The aim of this study was to evaluate whether diabetes mellitus, obesity and overweight are risk factors for the short-term post-operative outcome after major liver resection.

**Methods:**

Four hundred seventeen major liver resections (≥ 3 segments) were selected from a prospective database. Exclusion criteria were prior liver resection in patient’s history and synchronous major intra-abdominal procedures. Overweight was defined as BMI ≥ 25 kg/m^2^ and < 30 kg/m^2^ and obesity as BMI ≥ 30 kg/m^2^. Primary end point was 90-day mortality and logistic regression was used for multivariate analysis. Secondary end points included morbidity, complications according to Clavien–Dindo classification, unplanned readmission, bile leakage, and liver failure. Morbidity was defined as occurrence of a post-operative complication during hospital stay or within 90 days postoperatively.

**Results:**

Fifty-nine patients had diabetes mellitus (14.1%), 48 were obese (11.6%) and 147 were overweight (35.5%). There were no statistically significant differences in mortality rates between the groups. In the multivariate analysis, diabetes was an independent predictor of morbidity (OR = 2.44, p = 0.02), Clavien–Dindo grade IV complications (OR = 3.6, p = 0.004), unplanned readmission (OR = 2.44, p = 0.04) and bile leakage (OR = 2.06, p = 0.046). Obese and overweight patients did not have an impaired post-operative outcome compared patients with normal weight.

**Conclusions:**

Diabetes has direct influence on the short-term postoperative outcome with an increased risk of morbidity but not mortality. Preoperative identification of high-risk patients will potentially decrease complication rates and allow for individual patient counseling as part of a shared decision-making process. For obese and overweight patients, major liver resection is a safe procedure.

## Background

The indication for liver resection has been expanded significantly in recent years. Simultaneously, the operation's safety and efficacy have improved [1–6]. Nowadays, increasing proportions of patients with advanced age, neo-adjuvant treatment and associated comorbidities undergo even extended liver resections [6, 7]. Complex liver resection with concomitant biliary or vascular resection is a standard surgical procedure as part of a multi-disciplinary treatment approach [6]. However, major hepatectomy has been associated with a higher risk of post-operative mortality, morbidity, and higher rates of post-hepatectomy liver failure (PHLF) [8–11]. In the latter, the quality of the parenchyma within the future liver remnant has a crucial influence on the postoperative outcome. With the tremendous increase of diabetes mellitus [12], obesity and overweight [13] during the last decades a new group of patients with parenchymal disorders within the liver gets into focus of liver surgeons. The prevalence of diabetes and obesity in the adult population have reached values of 13% [14] and 37% [15] in the United States. Recent data clearly show that diabetes and obesity are frequently associated with non-alcoholic fatty liver disease (NAFLD) [16, 17] and may also be predictors for progression to fibrosis and cirrhosis [18, 19]. NAFLD was found to be associated with higher rates of postoperative mortality [20, 21], morbidity [21], PHLF [22] as well as infectious complications [23] after liver resection. However, influence of diabetes and obesity on the outcome after major liver resection is discussed controversially. While some authors showed higher rates of major complications in obese patients after major liver resection [17, 24] and higher mortality rates in morbidly obese patients after liver resection [25], others could not confirm those findings [26–29]. The evidence for the influence of diabetes on the outcome after liver resection is even weaker. Few studies have reported on the effect of diabetes on mortality after major liver resection [30, 31]. Here, the results were heterogeneous, too. Nevertheless, sometimes smaller centers even discourage diabetic and obese patients from potentially life-saving major liver resections out of fear of fatal outcomes. An accurate assessment of the safety of major liver resections by clear-cut definition of risk groups will enable those surgeons to provide appropriate counseling to their patients within a shared decision-making process. The 90-day mortality rate as well as classification of complications according to Clavien–Dindo [32] and the unplanned readmission rate are broadly accepted reliable surrogates for the short-term outcome after hepatectomy [26, 33–35].

Aim of the study was to evaluate the influence of diabetes mellitus, obesity and overweight on the short-term outcome after major hepatectomy.

## Methods

All patients undergoing major hepatectomy were considered for inclusion. The prospective liver resection database of the institution represented more than 99% of all performed liver resections of the department. Additional information was acquired retrospectively from the patient files and missing data on the postoperative course were obtained by contacting the patients, physicians or registration offices. Patients younger than 18 years of age, patients with antecedent liver resection, with in-situ split or laparoscopic resection, with liver resections performed simultaneously with other operations (such as pancreaticoduodenectomy, the unroofing of simple or parasitic cysts, cystectomy, and necrosectomy) as well as patients with hepatobiliary trauma or resection after liver transplantation were excluded from the analysis. Major liver resection was defined as resection of three or more Couinaud segments and the extent of liver resection was described according to the Brisbane 2000 system [36, 37]. Analysis of the data was approved by the ethical review committee of the University of Heidelberg (07/2013).

The indication for surgical treatment was confirmed by a multidisciplinary team by evaluation of each individual case within the weekly liver surgery tumor board. The extent of surgery depended on the preoperative presumptive diagnosis, the extent of tumor, the liver function, and other factors. In dependence of the characteristics of the parenchyma and the preference of the surgeon the liver parenchyma was transected using the vascular stapler, LigaSure™, clamp-crushing technique or Cavitron Ultrasonic Surgical Aspirator (CUSA). Portal triad clamping and selective inflow occlusion were performed as necessary in a minority of procedures. The central venous pressure was reduced to ≤ 5 mmHg during the parenchymal transection.

According to their body mass index (BMI), patients were divided into the following groups, which represent a modification of the WHO classification [38]: Underweight < 18.5, 18.5 ≤ normal weight < 25, 25 ≤ overweight < 30, obesity ≥ 30, and morbid obesity ≥ 35 kg/m^2^. Data on diabetes mellitus and other comorbidities were based on diagnoses reported in the health record after thorough preoperative exploration. Diabetes mellitus included diabetes mellitus type 1 as well as type 2. The laboratory results, that were as close as possible to the date of surgery, but not older than 30 days were registered. Tumor diagnosis was confirmed postoperatively by histopathological examination.

Complications were recorded and graded according to the Clavien-Dindo (CD) classification [32]. The highest grade was registered for each patient and CD grade IV was evaluated in further analysis. Morbidity was defined as the occurrence of a complication during the initial hospital stay or within 90 postoperative days. It included bile leakage, post-hepatectomy hemorrhage and PHLF all defined as proposed by the International Study Group of Liver Surgery (ISGLS) [39–41], wound infection, wound healing disorder, wound dehiscence, intra-abdominal infection, liver abscess, cholangitis, urinary tract infection, central line infection, sepsis, atelectasis, pneumonia, pleural effusion, pleural empyema, pulmonary embolism, respiratory decompensation, multiple organ failure, myocardial infarction, cardiac arrhythmia, acute renal failure, gastrointestinal bleeding, and thrombotic complications. Unplanned readmission was defined as readmission to any hospital within 90 days after discharge due to a complication related to surgery.

### Endpoints

The primary endpoint of the study was the 90-day mortality. Secondary endpoints were 30-day mortality, morbidity, Clavien–Dindo grade IV complications, unplanned readmissions, bile leakage, post-hepatectomy hemorrhage and post-hepatectomy liver failure (PHLF). Short-term outcome encompassed these endpoints. Exposure variables were diabetes mellitus, obesity and overweight.

### Statistics

Statistical analysis was performed using the software R (Version 3.2.2) [42]. Univariate analysis was performed using Pearson's chi-square test with Yates' continuity correction. Two-tailed Fisher's exact test was used instead when the expected cell count in any cell of the chi-square test was below five. Normal weight was used as reference for overweight and obesity. Multivariate analysis using logistic regression was carried out to assess whether diabetes mellitus, overweight, and obesity were independent predictors of a worse short-term outcome. Preoperative and operative characteristics that were significant for mortality in univariate analysis were chosen as covariates (data not shown). With this approach the following variables were included: Age of 60 years or more at the day of surgery, male gender, arterial hypertension, chronic renal failure, preoperative chemotherapy, right trisectionectomy, left trisectionectomy, biliodigestive anastomosis, benign indication, colorectal liver metastasis (CRLM) and cholangiocarcinoma. The results of the multivariate analysis were expressed as odds ratio (OR) and 95% confidence interval (95% CI). A p-value ≤ 0.05 was considered significant.

## Results

**Fig. 1:**
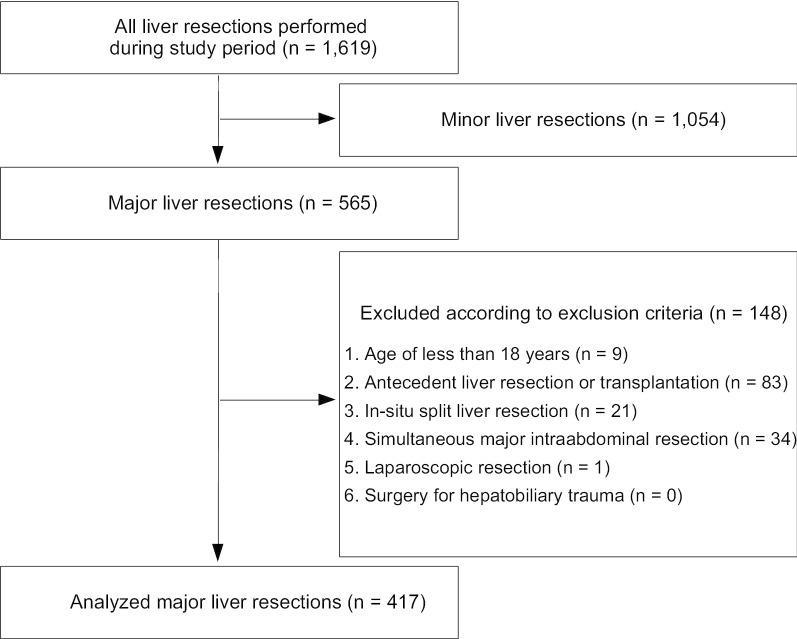
Participant flow diagram showing the patient selection process

The database included 1619 liver resections, 565 of which were major liver resections. After applying the exclusion criteria 417 patients were included into the analysis. The participant flow diagram is depicted in Fig. 1.

The preoperative patient characteristics, surgical procedures, and histopathological results as well as their associations with diabetes are summarized in Table 1 and their associations with overweight, and obesity are summarized in Table 2. Patients with diabetes had more frequently an age ≥ 60 years, male gender, ASA classification ≥ III, arterial hypertension, and hepatocellular carcinoma than non-diabetics (p-values < 0.0001, 0.003, 0.001, < 0.0001, and 0.01, respectively). Diabetics were less likely to receive preoperative chemotherapy and to be operated for CRLM (p-values 0.01 and 0.03, respectively). Patients with overweight were more frequently male and had higher rates of arterial hypertension and liver steatosis of ≥ 5% than patients without overweight (p-values 0.04, 0.0002, and 0.004, respectively). Obese patients were more likely to have arterial hypertension, cardiac failure, and liver steatosis of ≥ 5% than non-obese patients (p-values < 0.0001, 0.01, and < 0.0001, respectively). Neither overweight nor obesity were associated with a special tumor type or with diabetes mellitus. Diabetes mellitus was not associated with overweight or obesity.

**Table 1:** Preoperative patients characteristics, surgical procedures, and histopathological results in association with diabetes mellitus

	Study population	Diabetes mellitus	
	n/N (%) or mean ± standard deviation	n (%)	p
Total	417/417 (100)	59 (14.1)	
*Pre-operative patient characteristics*			
Body mass index (kg/m^2^), n = 414	25.2 ± 4.1		
Underweight	15/414 (3.6)	2 (3.4)	1
Normal weight	204/414 (49.3)	22 (37.9)	0.09
Overweight	147/414 (35.5)	27 (46.6)	0.08
Obesity	48/414 (11.6)	7 (12.1)	1
Morbid obesity	9/414 (2.2)	1 (1.7)	1
Age (years), n = 417	59.1 ± 13.4		
Age ≥ 60 years	226/417 (54.2)	48 (81.4)	** < 0.0001**
Male gender	232/417 (55.6)	44 (74.6)	**0.003**
ASA classification, n = 410	2.4 ± 0.6		
ASA III classification or more	192/410 (46.8)	39 (67.2)	**0.001**
Pre-existing disease			
Arterial hypertension	184/417 (44.1)	50 (84.7)	** < 0.0001**
Cardiac failure	7/417 (1.7)	3 (5.1)	0.06
Chronic renal failure	16/417 (3.8)	3 (5.1)	0.48
Lung disease	33/417 (7.9)	3 (5.1)	0.6
Hepatitis B or C	18/417 (4.3)	1 (1.7)	0.49
Esophageal varices	4/417 (1)	0 (0)	1
Regular alcohol consumption	28/417 (6.7)	4 (6.8)	1
Nicotine abuse	69/417 (16.5)	8 (13.6)	0.63
Pre-operative treatment			
Chemotherapy	106/417 (25.4)	6 (10.2)	**0.01**
Chemoembolization	11/417 (2.6)	0 (0)	0.38
Portal vein embolization	12/417 (2.9)	4 (6.8)	0.07
Laboratory values			
International normalized ratio > 1.2	11/416 (2.6)	1 (1.7)	1
Total bilirubin > 1 mg/dl	80/411 (19.5)	13 (22.4)	0.66
*Characteristics of surgery*			
Extent of surgery			
Right hemihepatectomy	226/417 (54.2)	31 (52.5)	0.89
Left hemihepatectomy	90/417 (21.6)	13 (22)	1
Right trisectionectomy	56/417 (13.4)	7 (11.9)	0.86
Left trisectionectomy	27/417 (6.5)	6 (10.2)	0.25
Segmental resection*	18/417 (4.3)	2 (3.4)	1
Adrenalectomy	12/417 (2.9)	0 (0)	0.23
Biliodigestive anastomosis	107/417 (25.7)	16 (27.1)	0.91
Resection device / technique			
Stapler	299/406 (73.6)	46 (83.6)	0.1
Ligasure	34/406 (8.4)	3 (5.5)	0.6
Clamp-crushing technique	35/406 (8.6)	2 (3.6)	0.2
Cavitron Ultrasonic Surgical Aspirator	32/406 (7.9)	4 (7.3)	1
Others	6/406 (1.5)	0 (0)	1
Pringle maneuver	88/411 (21.4)	12 (21.1)	1
Operative time (min), n = 410	237.7 ± 110		
Blood loss ≥ 1000 ml, n = 409	148/409 (36.2)	26 (44.8)	0.18
Transfusion of packed red blood cells	148/417 (35.5)	28 (47.5)	0.054
*Characteristics of histopathology*			
Non-malignant indication	57/417 (13.7)	3 (5.1)	–
Living liver donation	8/417 (1.9)	0 (0)	0.61
Malignant indication	360/417 (86.3)	56 (94.9)	0.06
Colorectal liver metastasis	123/417 (29.5)	10 (16.9)	**0.03**
Other liver metastases	56/417 (13.4)	9 (15.3)	0.81
Cholangiocarcinoma	136/417 (32.6)	25 (42.4)	0.12
Hepatocellular carcinoma	37/417 (8.9)	11 (18.6)	**0.01**
Other malignant tumor	8/417 (1.9)	1 (1.7)	1
Tumor diameter > 2.5 cm	268/369 (72.6)	45 (81.8)	0.14
Liver cirrhosis	15/417 (3.6)	4 (6.8)	0.25
Liver steatosis ≥ 5%	155/417 (37.2)	28 (47.5)	0.11

Bold values represent statistically significant results (*p*-values < 0.05)

*ASA* American Society of Anesthesiologists. Binary variables are given as frequency (proportion) in all columns except for the column 'Study population' where the number of patients without missing data for this variable is given additionally. Continuous variables are given as mean ± standard deviation

*≥3 segments not classified by formal terms such as hemihepatectomy or trisectionectomy

**Table 2:** Preoperative patients characteristics, surgical procedures, and histopathological results in association overweight, and obesity

	Study population	Normal weight	Overweight		Obesity	
	n/N (%) or mean ± standard deviation	n (%)	n (%)	p†	n (%)	p†
Total	417/417 (100)	204 (49.3)	147 (35.5)		48 (11.6)	
*Pre-operative patient characteristics*						
Diabetes mellitus	59/417 (14.1)	22 (10.8)	27 (18.4)	0.06	7 (14.6)	0.62
Age (years), n = 417	59.1 ± 13.4					
Age ≥ 60 years	226/417 (54.2)	105 (51.5)	90 (61.2)	0.09	23 (47.9)	0.78
Male gender	232/417 (55.6)	108 (52.9)	95 (64.6)	**0.04**	26 (54.2)	1
ASA classification, n = 410	2.4 ± 0.6					
ASA III classification or more	192/410 (46.8)	84 (41.8)	72 (50.3)	0.14	27 (56.3)	0.1
Pre-existing disease						
Arterial hypertension	184/417 (44.1)	67 (32.8)	78 (53.1)	**0.0002**	33 (68.8)	** < 0.0001**
Cardiac failure	7/417 (1.7)	1 (0.5)	2 (1.4)	0.57	4 (8.3)	**0.01**
Chronic renal failure	16/417 (3.8)	4 (2)	9 (6.1)	0.08	2 (4.2)	0.32
Lung disease	33/417 (7.9)	13 (6.4)	12 (8.2)	0.66	4 (8.3)	0.54
Hepatitis B or C	18/417 (4.3)	8 (3.9)	6 (4.1)	1	3 (6.3)	0.44
Esophageal varices	4/417 (1)	3 (1.5)	1 (0.7)	0.64	0 (0)	1
Regular alcohol consumption	28/417 (6.7)	11 (5.4)	10 (6.8)	0.75	5 (10.4)	0.2
Nicotine abuse	69/417 (16.5)	30 (14.7)	25 (17)	0.66	6 (12.5)	0.87
Pre-operative treatment						
Chemotherapy	106/417 (25.4)	52 (25.5)	36 (24.5)	0.93	11 (22.9)	0.85
Chemoembolization	11/417 (2.6)	7 (3.4)	3 (2)	0.53	1 (2.1)	1
Portal vein embolization	12/417 (2.9)	10 (4.9)	2 (1.4)	0.13	0 (0)	0.22
Laboratory values						
INR > 1.2	11/416 (2.6)	7 (3.4)	3 (2)	0.53	0 (0)	0.35
Total bilirubin > 1 mg/dl	80/411 (19.5)	43 (21.3)	28 (19.3)	0.75	6 (12.8)	0.26
*Characteristics of surgery*						
Extent of surgery						
Right hemihepatectomy	226/417 (54.2)	110 (53.9)	77 (52.4)	0.86	25 (52.1)	0.95
Left hemihepatectomy	90/417 (21.6)	43 (21.1)	34 (23.1)	0.74	12 (25)	0.69
Right trisectionectomy	56/417 (13.4)	31 (15.2)	17 (11.6)	0.41	6 (12.5)	0.8
Left trisectionectomy	27/417 (6.5)	13 (6.4)	10 (6.8)	1	4 (8.3)	0.54
Segment resection*	18/417 (4.3)	7 (3.4)	9 (6.1)	0.35	1 (2.1)	1
Adrenalectomy		5 (2.5)	3 (2)	1	4 (8.3)	0.07
Biliodigestive anastomosis		55 (27)	41 (27.9)	0.94	8 (16.7)	0.19
Resection device / technique						
Stapler		142 (72.1)	108 (74.5)	0.71	35 (76.1)	0.71
Ligasure		20 (10.2)	10 (6.9)	0.39	2 (4.3)	0.27
Clamp-crushing technique		14 (7.1)	15 (10.3)	0.39	6 (13)	0.23
CUSA		17 (8.6)	10 (6.9)	0.71	3 (6.5)	0.77
Others		4 (2)	2 (1.4)	1	0 (0)	1
Pringle maneuver		41 (20.5)	30 (20.5)	1	11 (23.4)	0.81
Operative time (min), n = 410						
Blood loss ≥ 1000 ml, n = 409		74 (37)	59 (41.3)	0.49	11 (22.9)	0.09
Transfusion of pRBC		70 (34.3)	57 (38.8)	0.46	15 (31.3)	0.81
*Characteristics of histopathology*						
Non-malignant indication		27 (13.2)	19 (12.9)	–	5 (10.4)	–
Living liver donation		5 (2.5)	3 (2)	1	0 (0)	0.59
Malignant indication		177 (86.8)	128 (87.1)	1	43 (89.6)	0.77
Colorectal liver metastasis		53 (26)	46 (31.3)	0.33	18 (37.5)	0.16
Other liver metastases		33 (16.2)	18 (12.2)	0.38	3 (6.3)	0.12
Cholangiocarcinoma		65 (31.9)	51 (34.7)	0.66	18 (37.5)	0.56
Hepatocellular carcinoma		22 (10.8)	10 (6.8)	0.28	3 (6.3)	0.43
Other malignant tumor		4 (2)	3 (2)	1	1 (2.1)	1
Tumor diameter > 2.5 cm		129 (71.7)	92 (71.3)	1	37 (78.7)	0.43
Liver cirrhosis		8 (3.9)	4 (2.7)	0.75	3 (6.3)	0.44
Liver steatosis ≥ 5%		55 (27)	62 (42.2)	**0.004**	35 (72.9)	** < 0.0001**

Bold values represent statistically significant results (*p*-values < 0.05)

†Normal weight was used as reference for overweight and obesity. Underweight is not shown due to the low number of patients (n = 15). *ASA* American Society of Anesthesiologists, *INR* International normalized ratio, *CUSA* Cavitron Ultrasonic Surgical Aspirator, *pRBC* packed red blood cells. Binary variables are given as frequency (proportion) in all columns except for the column 'Study population' where the number of patients without missing data for this variable is given additionally. Continuous variables are given as mean ± standard deviation

*≥3 segments not classified by formal terms such as hemihepatectomy or trisectionectomy

In univariate analysis Diabetes mellitus was associated with significantly higher rates of 30- and 90-day mortality, morbidity, CD grade IV, and PHLF (p-values 0.02, 0.03, 0.01, 0.0004, and 0.01, respectively). In addition, diabetic patients had significantly higher rates of wound healing disorders (35.7% in diabetics versus 20% in non-diabetics, p = 0.01), pneumonia (17.9% vs. 8.4%, p = 0.048), pleural effusion (35.7% vs. 16.7%, p = 0.002), respiratory decompensation (25% vs. 5.7%, p < 0.0001), acute renal failure (25% vs. 7.2%, p < 0.0001), and gastrointestinal bleeding (8.9% vs. 1.2%, p = 0.004). Overall 30- and 90-day mortality after major resection was 6.1% (n = 25) and 11.8% (n = 47), respectively, and overall-morbidity rate including CD I to V complications was 59.8% (234 patients). Complications of CD grade IV occurred in 10.1% (n = 42) of patients, and 19.7% (n = 68) of patients had unplanned readmissions. Bile leakage ISGLS Grade A to C was recorded in 23.8% (n = 93), post-hepatectomy hemorrhage ISGLS Grade A to C in 5.1% (n = 20) and PHLF ISGLS Grade A to C in 18.2% (n = 71).

Table 3 shows the results of the multivariate analysis. Diabetes mellitus was an independent predictor of higher rates of morbidity (OR = 2.44, p = 0.02), CD grade IV complications (OR = 3.6, p = 0.004), unplanned readmissions (OR = 2.44, p = 0.04), and bile leakage (OR = 2.06, p = 0.046). The influence of diabetes mellitus on 30- and 90-day mortality and PHLF could not be confirmed in multivariate analysis. In accordance with univariate analysis, neither overweight nor obesity were associated with any of the outcome variables. The results of the multivariate analysis for the other independent variables are shown in the Additional file 1: Table S1.

**Table 3:** Multivariate analysis of association of preoperative diabetes mellitus, overweight, and obesity with postoperative outcome variables

		Diabetes mellitus		Overweight		Obesity	
	n	OR (95%CI)	p	OR (95%CI)	p	OR (95%CI)	p
30-day mortality	405	1.82 (0.6; 5.47)	0.29	0.36 (0.12; 1.08)	0.07	0.47 (0.09; 2.49)	0.38
90-day mortality	395	1.22 (0.5; 2.95)	0.66	0.85 (0.39; 1.86)	0.69	0.48 (0.12; 1.94)	0.3
Morbidity	389	2.44 (1.15; 5.19)	**0.02**	1.46 (0.87; 2.43)	0.15	1.06 (0.49; 2.28)	0.89
Clavien–Dindo grade IV	414	3.6 (1.49; 8.66)	**0.004**	1.56 (0.73; 3.35)	0.25	1.76 (0.58; 5.36)	0.32
Unplanned readmission rate	342	2.44 (1.03; 5.78)	**0.04**	1 (0.53; 1.87)	0.99	1.07 (0.41; 2.76)	0.89
Bile leakage	389	2.06 (1.01; 4.21)	**0.046**	1.42 (0.8; 2.5)	0.23	1.46 (0.62; 3.41)	0.38
Posthepatectomy haemorrhage	389	0.73 (0.17; 3.03)	0.66	1.4 (0.5; 3.93)	0.52	1.98 (0.44; 8.94)	0.38
Posthepatectomy liver failure	389	1.76 (0.81; 3.82)	0.15	1.63 (0.86; 3.11)	0.13	0.54 (0.16; 1.82)	0.32

Bold values represent statistically significant results (*p*-values < 0.05)

*OR* odds ratio, *95% CI* 95% confidence interval. Results for the following model variables are shown in the supplementary appendix: underweight, age ≥ 60 years, male gender, arterial hypertension, chronic renal failure, preoperative chemotherapy, extended right hemihepatectomy, extended left hemihepatectomy, biliodigestive anastomosis, benign indication, colorectal liver metastasis and cholangiocarcinoma. The endpoints bile leakage, post-hepatectomy haemorrhage and post-hepatectomy liver failure were defined as proposed by the International Study Group of Liver Surgery [27–29]

## Discussion

It is discussed controversially, whether diabetes mellitus, obesity, and overweight are major risk factors for the short-term outcome after liver resection. The present study analyzed the patient population of a tertiary referral center with a high caseload of complex major liver resections.

Diabetes mellitus was found to independently predict a complicated postoperative course including significantly higher rates of morbidity, major complications, unplanned readmissions, and bile leakages, but it was not independently associated with a higher mortality rate. The present results are supported by the meta-analysis of Li et al., which showed higher rates of postoperative morbidity, liver failure, and infectious complications in diabetic patients [43]. Their study did not differentiate between major and minor resections, however. Few studies have analyzed the risk of diabetic patients after major liver resection previously. They reported heterogeneous results and often included only one histopathological entity. The results of Poon et al. in patients with hepatocellular carcinoma support the findings of the present analysis: diabetic patients did not have an increased risk for mortality after major resection [31]. In contrast, Little et al., showed different results for patients undergoing liver resection for CRLM. They found that diabetes went along with a higher mortality and no higher morbidity [30]. However, the comparability to the present study might be limited because in the study of Little et al., all diabetic patients that died after major liver resection had received neo-adjuvant chemotherapy. Balzan et al. analyzed the impact of overweight on the outcome after hepatectomy. Diabetes was not an independent predictor of major postoperative complications, but a detailed subgroup analysis for diabetic patients was not included [33]. In consideration of the present findings, diabetic patients should be informed about a higher risk of a complicated postoperative course, which warrants increased alertness and an experienced postoperative care setting. Nevertheless, as diabetes mellitus was not an independent predictor of mortality, these patients should not be denied major liver resection. Given the results of Little et al. even higher precaution might be necessary for patients with diabetes, that received preoperative chemotherapy.

In contrast to the higher risk of patients with diabetes, the present study found no higher mortality and morbidity rates in patients with obesity or overweight. These results are supported by the findings of Mathur et al. [44] and Viganò et al. [26], who showed no independent association of obesity and overweight with mortality and morbidity after major liver resection. However, there are heterogeneous reports in the literature. An increased risk for major complications after major resections was reported in obese and overweight patients previously [24, 33]. Since mortality rates were not increased in those reports, this should not be considered a limitation for surgery [33]. The study of Zogg et al. found only morbid obesity to be associated with higher mortality and morbidity rates, while non-morbid obesity and overweight were no risk factors [25]. Similarly, the meta-analysis of Rong et al. on liver resection for HCC found no association between BMI and mortality [45]. In accordance with the findings of the present study, there is no reason to deprive overweight patients of major liver resections. Nevertheless, the subgroup of morbidly obese patients should be assessed with special attention.

The current study found diabetes to be associated with several individual complications. The most notable were bile leakage, pneumonia, respiratory insufficiency, acute renal failure, and gastrointestinal bleeding, which are potentially life-threatening [46, 47] and thus correspond to the higher rate of CD grade IV complications. The findings are supported by previous studies that found diabetics to suffer more frequently from infectious [48] and pulmonary [49] complications, and acute renal failure [27, 50, 51] after liver resection. They are in line with the detrimental effect of diabetes mellitus on immunological [52–54] and renal function [55]. The higher rates of bile leakage in diabetic patients are more difficult to understand. Potentially, the diabetic affection of the microcirculation [56] provoked biliary transudation and impaired healing at the resection surface.

Major liver resection is still associated with relevantly higher rates of complications and mortality than minor liver resection [8, 10, 11, 57]. In the present study, PHLF occurred in 18.2% of patients. Two recent studies on major liver resection that also applied the ISGLS definition found a PHLF rate of 9.6–30.1% [58, 59]. In the present cohort, 90-day mortality was 11.8%. This is within the numbers reported from hepatobiliary centers all over Germany [57]. While the analysis of major resections for CRLM showed 90-day mortality rates between 2 and 8% [60–63], 90-day mortality rates for perihilar cholangiocarcinoma of up to 14% [64] and up to 18% for HCCs have been reported [65]. Furthermore, plain major resections such as hemi-hepatectomies show a better outcome than extended liver resections [35, 62, 66]. In extended liver resections a 90-day mortality rate of up to 16.7% has been reported [67, 68].

The present study has some limitations. First, some diabetic, obese, or overweight patients might not have been presented to the surgeons as candidates for resection as their treating doctors might have considered them at high risk for a fatal postoperative outcome. Nevertheless, the analysis showed that neither diabetic, obese nor overweight patients received different extents of surgery compared to non-diabetic and normal weight patients, respectively. Second, diabetic patients less frequently underwent preoperative chemotherapy than non-diabetics. However, this was probably partly secondary to a significantly lower rate of CRLM among diabetic patients as the majority (i.e. 71.7%) of patients with preoperative chemotherapy had CRLM. Third, since the cohort included only patients from a European center the outcome might not be comparable with Asian cohorts where the BMI of diabetic patients is often normal and there has been not such a tremendous increase in the average BMI of the population [69].

## Conclusions

Diabetes mellitus is an independent risk factor for a complicated postoperative course after major liver resection with significantly higher rates of morbidity, major complications, unplanned readmissions, and bile leakages. However, it was not associated with a higher mortality rate. Individual patient counselling should be intensified for diabetics before major liver resection and extended resections should be performed in an experienced tertiary care center. In contrast, the data suggest obese and overweight patients to be safe to undergo major liver resection as they had no significantly increased postoperative mortality nor morbidity.

## Supplementary information

**Additional file 1: Table S1.** Multivariate logistic regression analysis of association of preoperative variables with postoperative outcome variables.

## Data Availability

Results of all multivariate analyses, which were performed in our study, are either presented in the manuscript or attached as supplementary material. All other data used for this study are presented in the tables attached.

## Abbreviations

BMI: Body Mass Index
CD: Clavien–Dindo Classification of surgical complications
CI: Confidence Interval
CRLM: Colorectal liver metastasis
CUSA: Cavitron ultrasonic aspirator
ISGLS: International Study Group of Liver Surgery
NAFLD: Non-alcoholic fatty liver disease
OR: Odds ratio
PHLF: Posthepatectomy liver failure
WHO: World Health Organization
