# Supplementary material for: Influence of diabetes on short-term outcome after major hepatectomy: an underestimated risk?
Source: BMC Surg. 2020 Nov 30;20:305. doi: 10.1186/s12893-020-00971-w (PMC7708157; doi:10.1186/s12893-020-00971-w)
Supplement: Supplementary file 1 — Additional file 1: Table S1. Multivariate logistic regression analysis of association of preoperative variables with postoperative outcome variables. [file 12893_2020_971_MOESM1_ESM.doc]

Table 1 Multivariate logistic regression analysis of association of preoperative variables with postoperative outcome variables.

† This calculation was not possible because this outcome occurred zero times in the patients with the given preoperative variable; OR, odds ratio; 95 %-CI, 95 % confidence interval. The data are given as odds ratio (2,5%-percentile; 97,5%-percentile).

Supplementary Data

Table 1 (continued) Multivariate logistic regression analysis of association of preoperative variables with postoperative outcome variables.

† This calculation was not possible because this outcome occurred zero times in the patients with the given preoperative variable; OR, odds ratio; 95 %-CI, 95 % confidence interval. The data are given as odds ratio (2,5%-percentile; 97,5%-percentile).
